# Supplementary material for: Experimental study platform for electrocatalysis of atomic-level controlled high-entropy alloy surfaces
Source: Nat Commun. 2023 Jul 26;14:4492. doi: 10.1038/s41467-023-40246-5 (PMC10372069; doi:10.1038/s41467-023-40246-5)
Supplement: Supplementary file 1 — Supplementary Information [file 41467_2023_40246_MOESM1_ESM.pdf]

Supplementary Information

Experimental Study Platform for Electrocatalysis of Atomic-level Controlled High-entropy Alloy Surfaces

Yoshihiro Chida*[1], Takeru Tomimori[1], Tomoaki Ebata[1], Noboru Taguchi[2], Tsutomu Ioroi[2], Kenta Hayashi[1], Naoto Todoroki[1] and Toshimasa Wadayama[1]

1: Graduate School of Environmental Studies, Tohoku University, Sendai 980-8579, Japan.

2: National Institute of Advanced Industrial Science and Technology, Ikeda 563-8577, Japan.

*; corresponding author

TEL: +81-22-795-7320

Email: yoshihiro.chida.t8@dc.tohoku.ac.jp

1. Details of XPS analysis and data processing

Table S1 Estimated alloy-composition ratios of Cr-Mn-Fe-Co-Ni layer deposited on Pt(111) without Pt following.

Element / band	Measured band area ratio* (%)	R.S.F.**	Composition ratio (%)
Cr 3*p*	15.26±0.41	1.00	20.07±0.76
Mn 3*p*	17.80±0.33	1.13	20.68±0.34
Fe 3*p*	18.49±0.36	1.23	19.82±0.45
Co 3*p*	21.55±0.21	1.41	20.15±0.27
Ni 3*p*	26.89±0.25	1.84	19.28±0.38

* Cr ~ Ni 3*p* area intensity ratios in APD depositing-layer with sufficient thickness, the average and the standard error of 5 measurements.

** Standardized with the value of Cr 3*p* as 1.00, evaluated by intensity on pure metal surfaces of each element.

Cr-Mn-Fe-Co-Ni/Pt(111), which is a 3-nm-thick Cr-Mn-Fe-Co-Ni (Cantor alloy[1]) layer, was deposited on a surface-cleaned Pt(111) substrate under ultra-high vacuum (UHV; < 10^{-7} Pa) and annealed in UHV at 773 K for 30 min. Then, the resulting Cr–Mn–Fe–Co–Ni/Pt(111) sample was stored in the transfer vessel[2] and vacuum-transferred to the XPS analysis system (see the Methods section in the main manuscript). A Mg Kα (1253.6 eV) X-ray was used as an X-ray photoelectron excitation source. The detection depth of the photoelectrons was approximately 3 nm[3], which almost corresponds to the deposited Cr-Mn-Fe-Co-Ni layer thickness. Because Cr-Mn-Fe-Co-Ni is a mixture of five neighbouring elements in the periodic table, estimations of the corresponding 2*p* band energy and band-area intensities of each element are generally difficult because each Auger electron peak tends to overlap with

the 2*p* bands, especially the 2*p* of Mn, Fe and Co. Therefore, instead of the 2*p* bands, the 3*p* bands (which appeared in the 70-35 eV binding energy regions) were used for the estimation. The 3*p* band energy was located considerably far from for the Auger electron peaks except for the overlap of the Pt 5*s* peak (approximately 51.0 eV).

Table S1 lists the ratios of the band areas of each 3*p* band of the deposited Cantor alloy layer in which the XPS system-specific relative sensitivity factors (R. S. F.) were separately evaluated from the 3 nm-thick single-constituent-element deposited samples (M/Pt(111), (M = Cr, Mn, Fe, Co, Ni)). The estimated alloy-composition ratios listed in Table S1 are the average of five XPS measurements with experimental errors. The results clearly indicate that the compositions of the deposited 3 nm-thick Cr-Mn-Fe-Co-Ni layer on Pt(111) were almost the same as those in the Cr-Mn-Fe-Co-Ni mixed APD target with an almost equi-atomic ratio of the constituent elements.

Pt/Cr-Mn-Fe-Co-Ni/Pt(111)

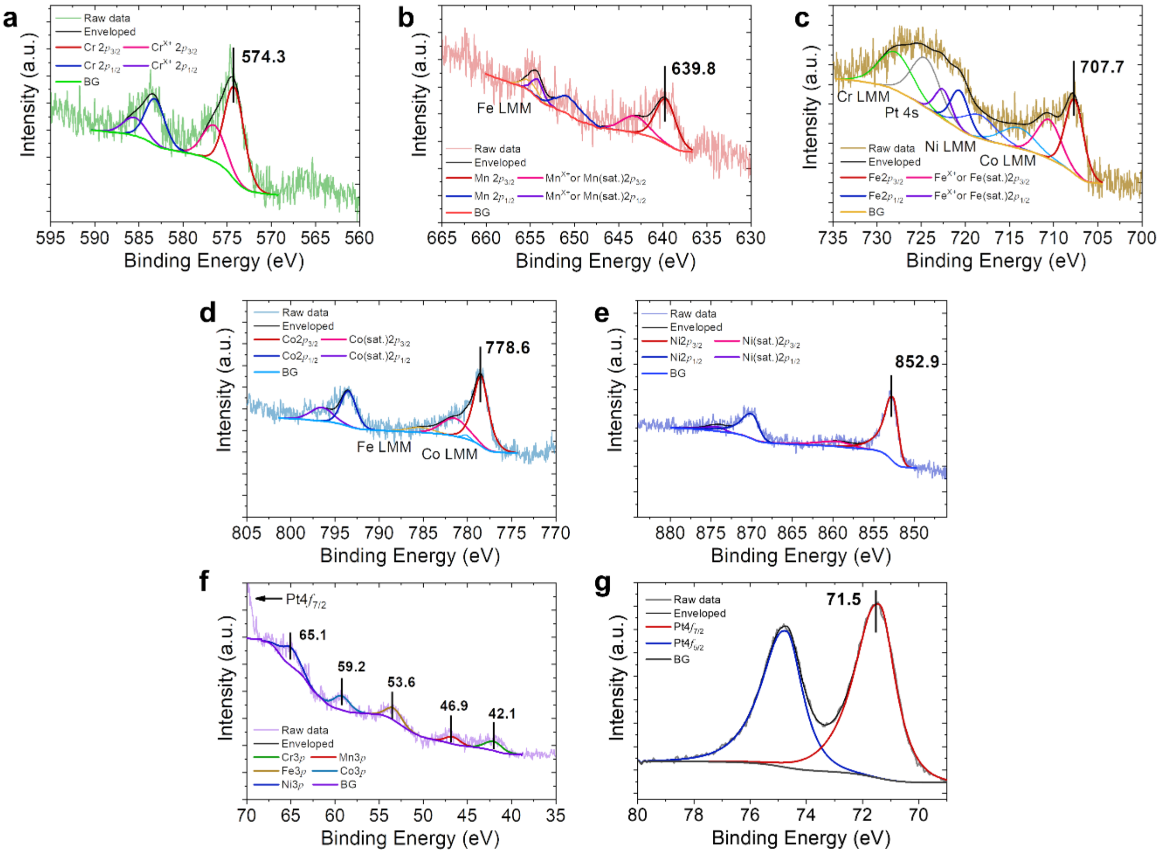

Fig. S1 XPS spectra of the (a) Cr 2*p* 595–560 eV, (b) Mn 2*p* 665–630 eV, (c) Fe 2*p* 735–700 eV, (d) Co 2*p* 805–770 eV, (e) Ni 2*p* 885–845 eV, (f) Cr to Ni 3*p* 70–35 eV and (g) Pt 4*f* 80–68 eV bands of the as-synthesised Pt/Cr-Mn-Fe-Co-Ni/Pt(111).

Pt/Co/Pt(111)

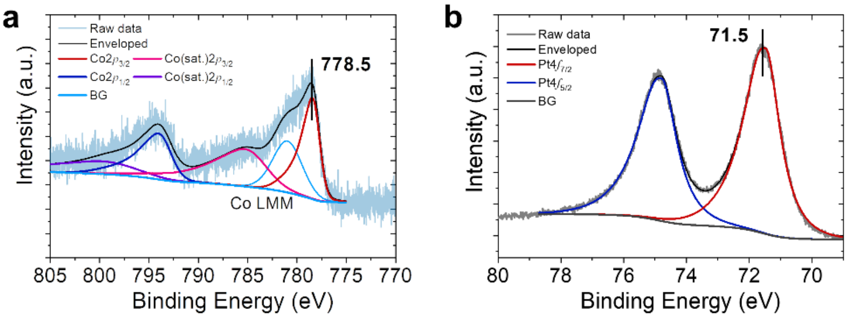

Fig. S2 XPS spectra of the (a) Co 2*p* (805–770 eV) and (b) Pt 4*f* (80–68 eV) bands of the as-synthesised Pt/Co/Pt(111).

Table S2 Peak position values of the higher spin-orbit-splitting side of the metallic state of each constituent element of the as-synthesised Pt/Cr-Mn-Fe-Co-Ni/Pt(111) and Pt/Co/Pt(111) compared with the values in the database library obtained from the National Institute of Standards and Technology (NIST)[4]. The unit is eV.

Element / band	Pt/Cr-Mn-Fe-Co-Ni/Pt(111)	Pt/Co/Pt(111)	Metallic state
Pt $4f_{7/2}$	71.5	71.5	71.5
Cr $2p_{3/2}$	574.3	———	574.2
Mn $2p_{3/2}$	639.8	———	639.3
Fe $2p_{3/2}$	707.7	———	707.1
Co $2p_{3/2}$	778.6		778.2
Ni $2p_{3/2}$	852.9	———	852.7
Cr $3p$	42.1	———	42.4
Mn $3p$	46.9	———	47.4
Fe $3p$	53.6	———	53.0
Co $3p$	59.2	59.4	59.7
Ni $3p$	65.1	———	66.2

Figs. S1 and S2 show the XPS spectra of the Cr to Ni $2p$, $3p$ and Pt $4f$ bands of the as-synthesised Pt/Cr-Mn-Fe-Co-Ni/Pt(111) and Pt/Co/Pt(111). The accuracy of the band energy was confirmed by calibrating the XPS system with surface-cleaned Cu and Au thin films prior to the measurements. Table S2 lists the summary of the band energy of the higher spin-orbit-splitting side (Cr $2p_{3/2}$, for example) of each metal state (oxidation number = 0) and the values of the corresponding pure elements obtained from the National Institute of Standards and Technology (NIST) database[4]. The band energy of the metallic states measured in the vacuum-synthesised samples were estimated using a typical resolution of less than 0.1 eV[5] by

deconvoluting each band according to the energy in each band. Auger electron peaks were also measured as listed in the database of the analysis software (Thermo Fisher Scientific, Avantage Data Analysis ver. 5, 973), a technical book that describes XPS analysis methods[6], and a recent report on XPS analysis of the Cantor alloy[7].

Generally, charge transfer from Pt to the alloying elements occurs in Pt-based binary alloys such as Pt–Co and Pt–Ni, which results in chemical shifts to the high-binding-energy sides of the Pt 4*f* band[8,9]. Numerous theoretical discussions on ORR-activity enhancement mechanisms by alloying have been reported[10–13]. The chemical shifts in the Pt 4*f* band energy caused by the charge transfer among the alloying elements were correlated with the activity-enhancement mechanisms. In this case, both Pt/Cr-Mn-Fe-Co-Ni/Pt(111) and Pt/Co/Pt(111) demonstrated a chemical shift to the high-energy side of the Pt 4*f* band with almost the same value (approximately 0.3 eV). The ORR activities of both samples were evaluated to be almost the same, approximately 11 times higher than that of clean Pt(111) (see the main manuscript).

2. Pt 4*f* band change before and after 5,000 PC-loadings

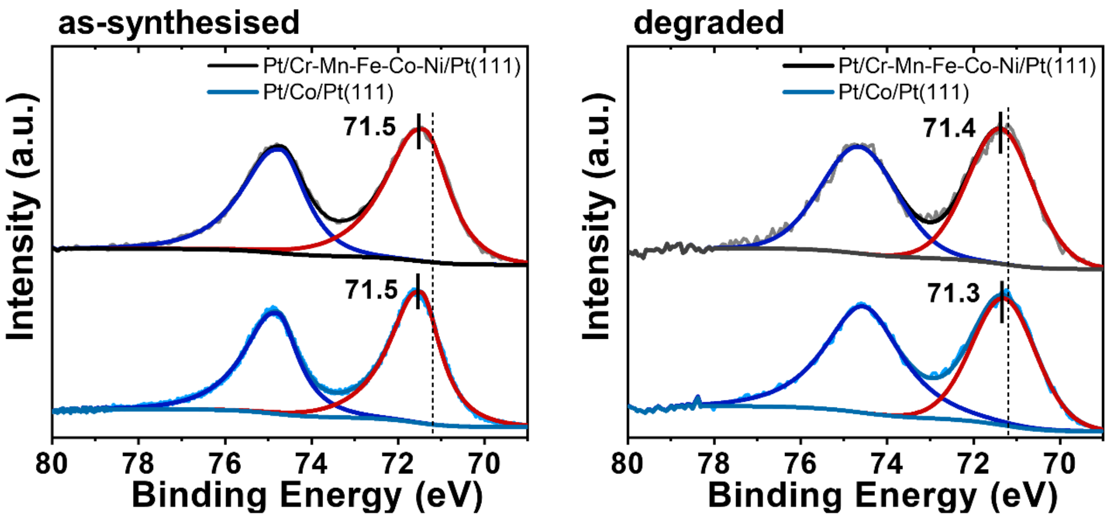

Fig. S3 Comparison of the Pt 4*f* bands of the Pt/Cr-Mn-Fe-Co-Ni/Pt(111) and Pt/Co/Pt(111) samples collected at the as-synthesised (0 PC-loading) and the degraded (5,000 PC-loadings) states.

Fig. S3 shows the collected (as-synthesised; left panel) and degraded (5,000 potential cycles (PC) loading; right panel) Pt 4*f* spectra of Pt/Cr-Mn-Fe-Co-Ni/Pt(111) (upper) and Pt/Co/Pt(111) (lower). Fig. S3 shows that the Pt 4*f* band energy of the as-synthesised samples and the corresponding estimated ORR-activity enhancement versus clean Pt(111) were almost the same (71.5 eV and 12 times higher activity than clean Pt(111), respectively (see main manuscript)). In contrast, the Pt4*f* of degraded Pt/Cr-Mn-Fe-Co-Ni/Pt(111) was located at approximately 0.1 eV at the higher energy side of Pt/Co/Pt(111), which indicated that the alloying elements worked more effectively even at the degraded state of Pt/Cr-Mn-Fe-Co-Ni/Pt(111) compared to that of Pt/Co/Pt(111) binary alloy system, resulted to the ORR enhancement caused by the charge transfer between Pt and underlaid Cantor alloy constituents.

3. Structural characterisation of Pt/Mn–Fe–Co–Ni/Pt(111)

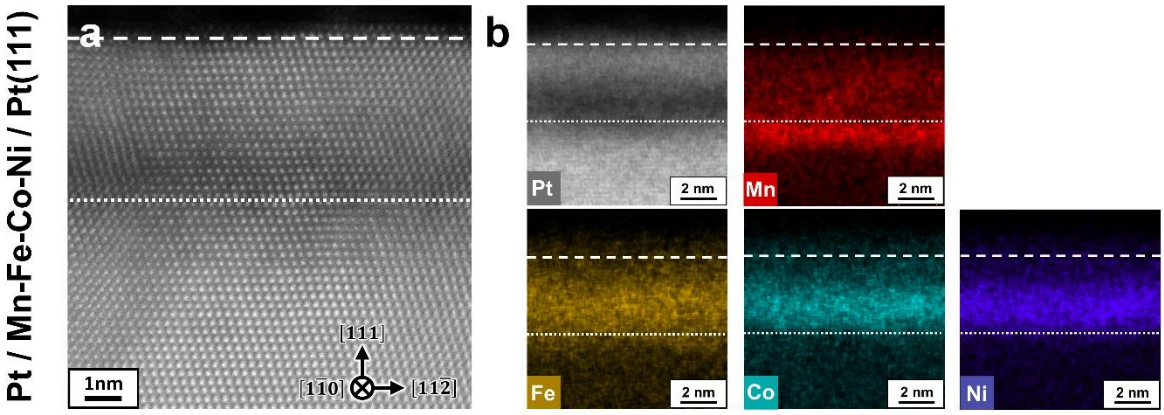

Fig. S4 As-synthesised surface structural characterisations of Pt/Mn-Fe-Co-Ni/Pt(*hkl*). a, b, Cross-sectional HAADF-STEM images (a) and colour-coded EDS-2D mapping with a 2-nm scale bar (b) of the as-synthesised Pt/Mn-Fe-Co-Ni/Pt(111).

The cross-sectional HAADF-STEM image and EDS mapping of the constituent elements of the as-synthesised Pt/Mn-Fe-Co-Ni/Pt(111) are shown in Figs. S4a and S4b, respectively. The vacuum-synthesis procedure was the same as that in Pt/Cr-Mn-Fe-Co-Ni/Pt(111) except for the APD target (an equi-atomic ratio Mn-Fe-Co-Ni alloy; see Methods section in the main manuscript). Fig. S4a shows that similar to Pt/Cr–Mn–Fe–Co–Ni/Pt(111), the epitaxial growth of the Mn–Fe–Co–Ni and surface Pt layers (the horizontal dashed and dotted lines are provided as eye guides for the original substrate and topmost surface, respectively) was clearly confirmed from the Pt(111) substrate to the topmost surface of Pt. We could notice that the Z-contrast of the STEM image (Fig. S4a) was more obscure than that of Pt/Cr-Mn-Fe-Co-Ni/Pt(111). The EDS mapping (Fig. S4b) indicated that the alloying elements were more broadly distributed on the surface stacking layers as well as in the substrate Pt than in Pt/Cr-Mn-Fe-Co-Ni/Pt(111). Mn was distributed on the Pt phase side rather than at the interface with the Pt substrate. The results demonstrated that because the same synthesis procedure was used, the thermal-diffusion of the constituent elements was more suppressed in the Pt/Cr-Mn-Fe-Co-Ni/Pt(111) six-element system than in the Pt/Mn-Fe-Co-Ni/Pt(111) five-

element system. This result could be attributed to the difference in the thermodynamic stability of the underlaid Cr-Mn-Fe-Co-Ni (Cantor alloy) and Mn-Fe-Co-Ni layers, e.g. difference in the degree of the so-called 'sluggish diffusion', which is one of the unique features of HEAs[14].

4. Atomic-level surface microstructures and ORR properties of Pt/Co/Pt(*hkl*)

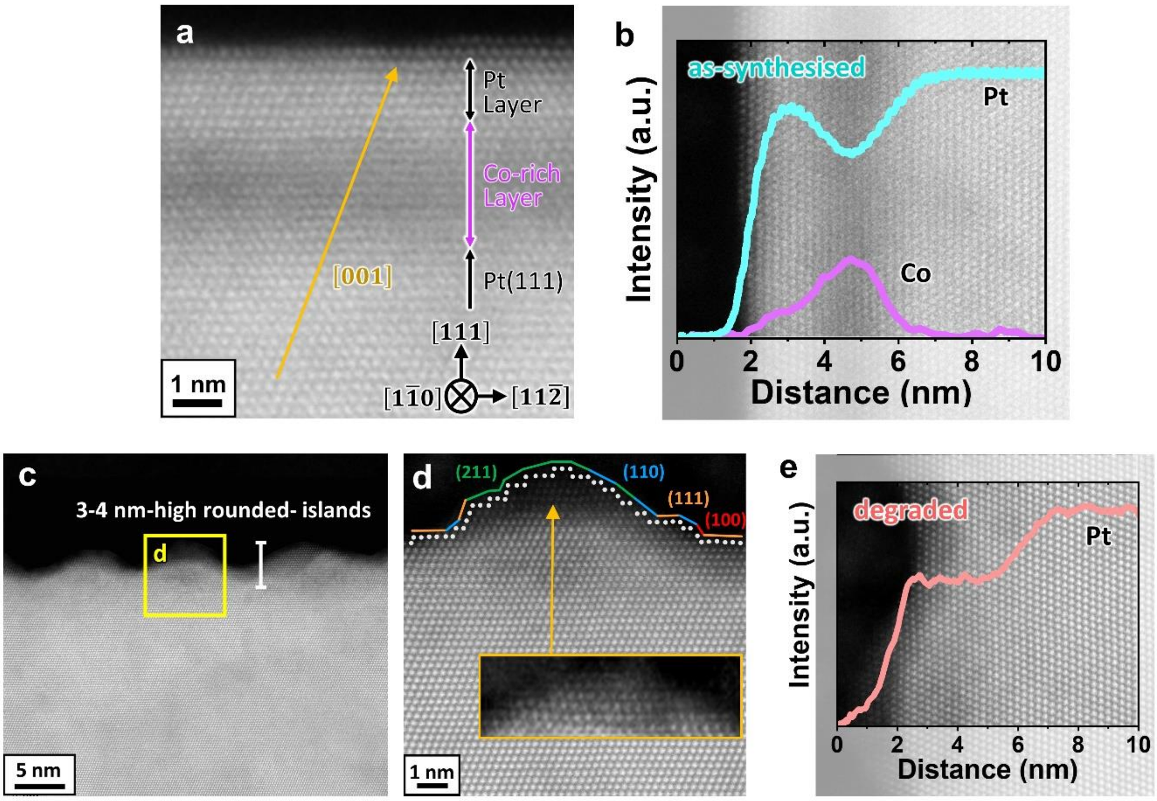

Fig. S5 As-synthesised and PC-loaded surface structure characterisation of Pt/Co/Pt(111). a, b, Cross-sectional HAADF-STEM images (a, c and e) and EDS intensity depth profiles of Pt (black) and Co (light blue) in the surface-normal direction of the as-synthesised Pt/Co/Pt(111). **c–e,** Cross-sectional HAADF-STEM images at relatively low magnification (c) and at atomically-resolved high-magnification of the marked regions indicated by the yellow square in (c) where the surface domains of (111), (110), (100) and (211) are highlighted in orange, blue, red and green, respectively. (d) EDS intensity depth profiles of Pt in the surface-normal direction of the 5,000 PC-loaded samples of Pt/Co/Pt(111).

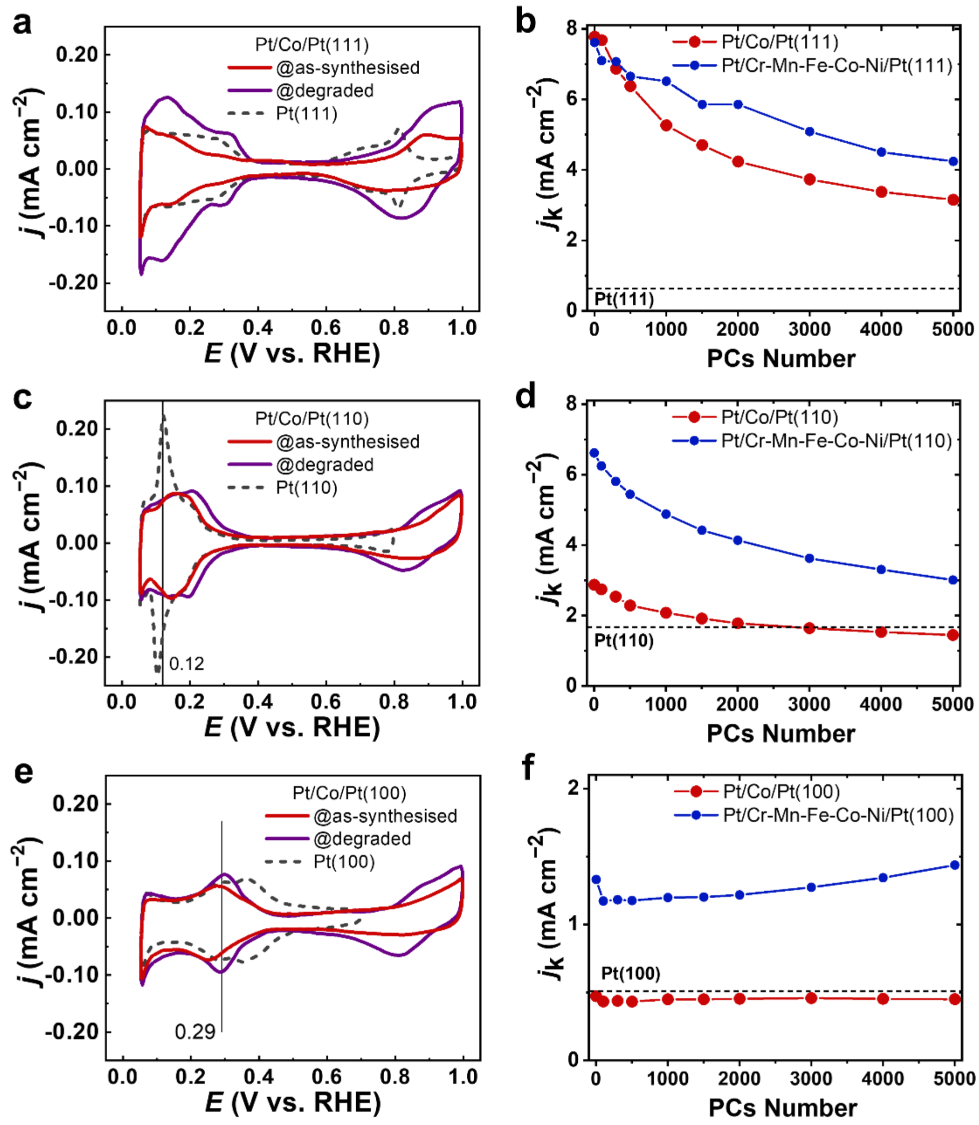

Fig. S6 CV curves and ORR activity trends under PC-loading of the vacuum-synthesised Pt/Co/Pt(*hkl*). a-f, Cyclic voltammetry (CV) curves collected at 0.05–1.0 V vs. reversible hydrogen electrode (RHE) potential range of the (red) as-synthesised and (purple) degraded states of Pt/Co/Pt(111), (110) and (100), respectively. Corresponding CV curves of the Pt(*hkl*) substrates are indicated by the dashed lines. (a, c and e) ORR activity trends recorded under PC-loading (up to 5,000 cycles at 0.6–1.0 V) of Pt/Co/Pt(111), (110) and (100), respectively. (b, d and f)

The cross-sectional atomic-level microstructures of the as-synthesised and 5,000 PC-loaded (degraded) Pt/Co/Pt(111) and the corresponding CV curves and the ORR-activity trends evaluated under PC-loading are shown in Figs. S5 and S6. The cross-sectional STEM image of the as-synthesised Pt/Co/Pt(111) shows that the underlaid Co and surface Pt(111) lattices

epitaxially grew on the Pt(111) substrate, and that stacking faults were absent in this field of view (Fig. S5a) Generally, the Co-rich interlayer is located between the surface Pt-rich(111) layer and substrate Pt(111) (Fig. S5b). However, the Z-contrast of the STEM image was obscure as compared to that in Pt/Cr-Mn-Fe-Co-Ni/Pt(111) (Fig. 2a in the main manuscript), which indicated that alloying of the deposited Co and subsequently deposited surface Pt occurred more actively through the UHV thermal annealing process (773 K for 30 min; see main manuscript). Regarding the surface microstructure of the degraded sample (5,000 PC-loadings), the atomically flat (111) surface (Fig. S5a) vanished and became rough (at a few nanometres level), forming a 3–4-nm-high island-like surface (Figs. S5c and S5d). In addition, the Z-contrast of the HAADF-STEM image of the surface Pt and underlaid Co-rich layers was almost absent. Furthermore, the EDS Pt signal-intensity changes in the surface-normal direction (Fig. S5e) were almost constant within approximately 4 nm of the topmost Pt surface after the PC-loading. These results suggest that Co tended to diffuse and segregate at the near-surface region and thus generated Pt-Co alloy layers, which were probably obtained by the oxygen potential[8,9,15] under an electrochemical environment (interface between the electrolyte and topmost Pt surface). Such surface segregation of the alloying element of Co during the PC-loading resulted in severe dissolution of Co into the electrolyte and structural degradation of the atomically flat Pt-enriched (111) original surface. The activity exhibited by Pt/Co/Pt(110) and (100) was clearly lower than that of Pt/Cr-Mn-Fe-Co-Ni/Pt(110) and (100), suggesting that the alloying effect was less pronounced in these surface orientations than in Pt-Cr-Mn-Fe-Co-Ni.

The electrocatalytic properties of Pt/Co/Pt(*hkl*) are described below. For the CV curves of the as-synthesised states, Pt/Co/Pt(*hkl*) exhibited typical CV curvess for the Pt-based alloy single-crystal surfaces[16,17]. The CV curve of as-synthesised Pt/Co/Pt(111) (red curve in Fig. S6a) shows onset-potential shifts in O/OH-related species adsorption (> 0.6 V) to a higher

potential than that of clean Pt(111) (dashed line). Regarding Pt/Co/Pt(110), the characteristic H adsorption/desorption response at approximately 0.12 V in Fig. S6c was completely absent, whereas that in Pt/Co/Pt(100) at approximately 0.29 V for clean Pt(100) in Fig. S6e was present. The CV curves of the degraded states (recorded after 5,000 PC-loadings) of Pt/Co/Pt(*hkl*) (purple) exhibited increases in H- (< 0.35 V) and O/OH- related species adsorption and desorption (> 0.6 V), which implies Pt surface roughening by the underlaid Co dissolution due to the PC-loading. With regard to the ORR-activity trends under the PC-loading (Figs. S6b, S6d and S6f), although the ORR initial activity of Pt/Co/Pt(111) was almost the same as that of Pt/Cr-Mn-Fe-Co-Ni/Pt(111) (Fig. 3b in the main manuscript), the activity trend evaluated under PC-loading was relatively steep, suggesting that more severe surface-structure degradations caused the ORR deactivation (Fig. S6b). Pt/Co/Pt(110) and (100) exhibited only half the initial activity of Pt/Cr-Mn-Fe-Co-Ni/Pt(110). Furthermore, ORR-activity enhancements against clean Pt(110) and (100) surfaces were almost absent in the degraded state (after 5,000 PC-loadings) (Figs. S6d and S6f).

5. iR-corrected LSV curves of Pt/Cr-Mn-Fe-Co-Ni/Pt(*hkl*) and Pt/Co/Pt(*hkl*).

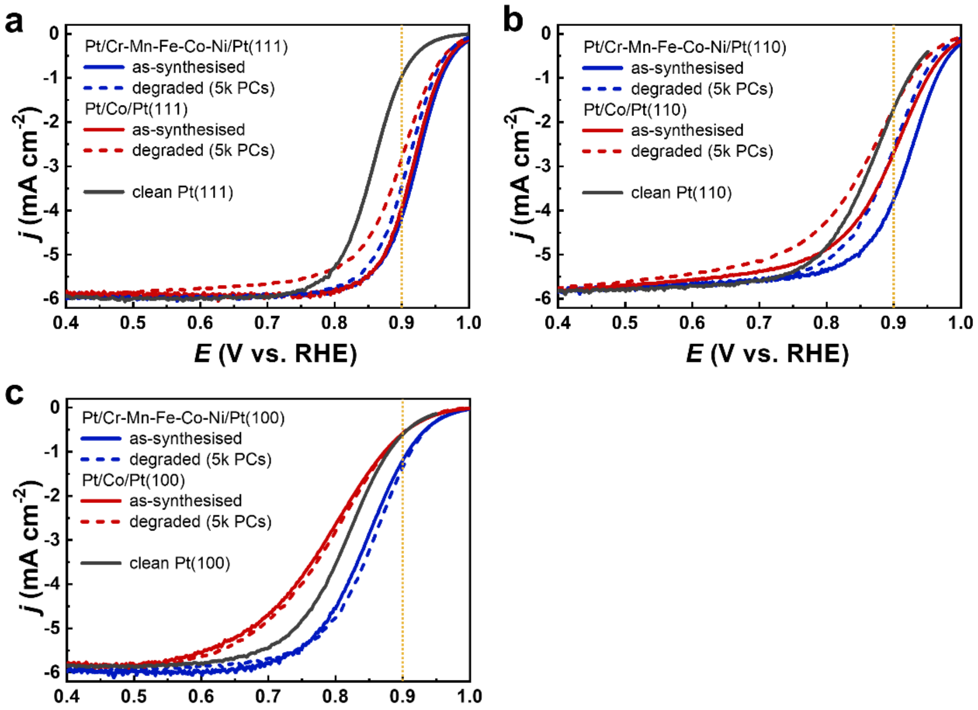

Fig. S7 iR-corrected LSV curves of the vacuum-synthesised Pt/Cr-Mn-Fe-Co-Ni/Pt(*hkl*) and Pt/Co/Pt(*hkl*). a-c, LSV curves collected at 0.05–1.05 V vs. RHE potential range of the as-synthesised (solid lines) and degraded (dashed lines) states of Pt/Cr-Mn-Fe-Co-Ni/Pt(*hkl*) (blue) and Pt/Co/Pt(*hkl*) (red) (*hkl* = 111, 110 and 100, respectively). The corresponding LSV curves of clean Pt(*hkl*) are indicated by the black solid lines. All current density values were iR-corrected by using 82.6 Ω as the solution resistance and the electrode resistance was small enough to ignore (<0.1 Ω)), determined by electrochemical impedance spectroscopy (EIS) measurement.

In this study, the ORR activity was generally evaluated by calculating the kinetic current values of ORR at 0.90 V vs. RHE estimated from the LSV curves measured with multiple rotational speeds by using the Koutecky-Levich equation[15,18,19] and dividing it by the geometric surface area of the electrode surface determined by the Karletz-made O-ring (0.0903 cm^2). The ORR activity trends shown in Figs. 3 in the main manuscript and in Figs. S6 are the results obtained without considering the iR drop of the electrochemical measurement system used in this study. Figs. S7a-S7c show the full-iR-corrected LSV curves of Pt/Cr-Mn-Fe-Co-Ni/Pt(*hkl*) and Pt/Co/Pt(*hkl*), respectively. iR was determined using the resistance estimated by

electrochemical impedance spectroscopy (EIS) for Pt/Cr-Mn-Fe-Co-Ni/Pt(111); the solution resistance was estimated to be 82.6 Ω and the electrode resistance was negligibly small (< 0.1 Ω). The ORR current densities (j) at 0.9 V vs. RHE for the full-iR-corrected LSV curves of the as-synthesised and degraded (PC-loaded) samples were within the potential range of the kinetic and diffusion mixed controlled region, indicating that ORR activity can be evaluated using the Koutecky-Levich equation[15,18,19].

6. CO stripping voltammetry for Pt/Cr-Mn-Fe-Co-Ni/Pt(*hkl*) and Pt/Co/Pt(*hkl*)

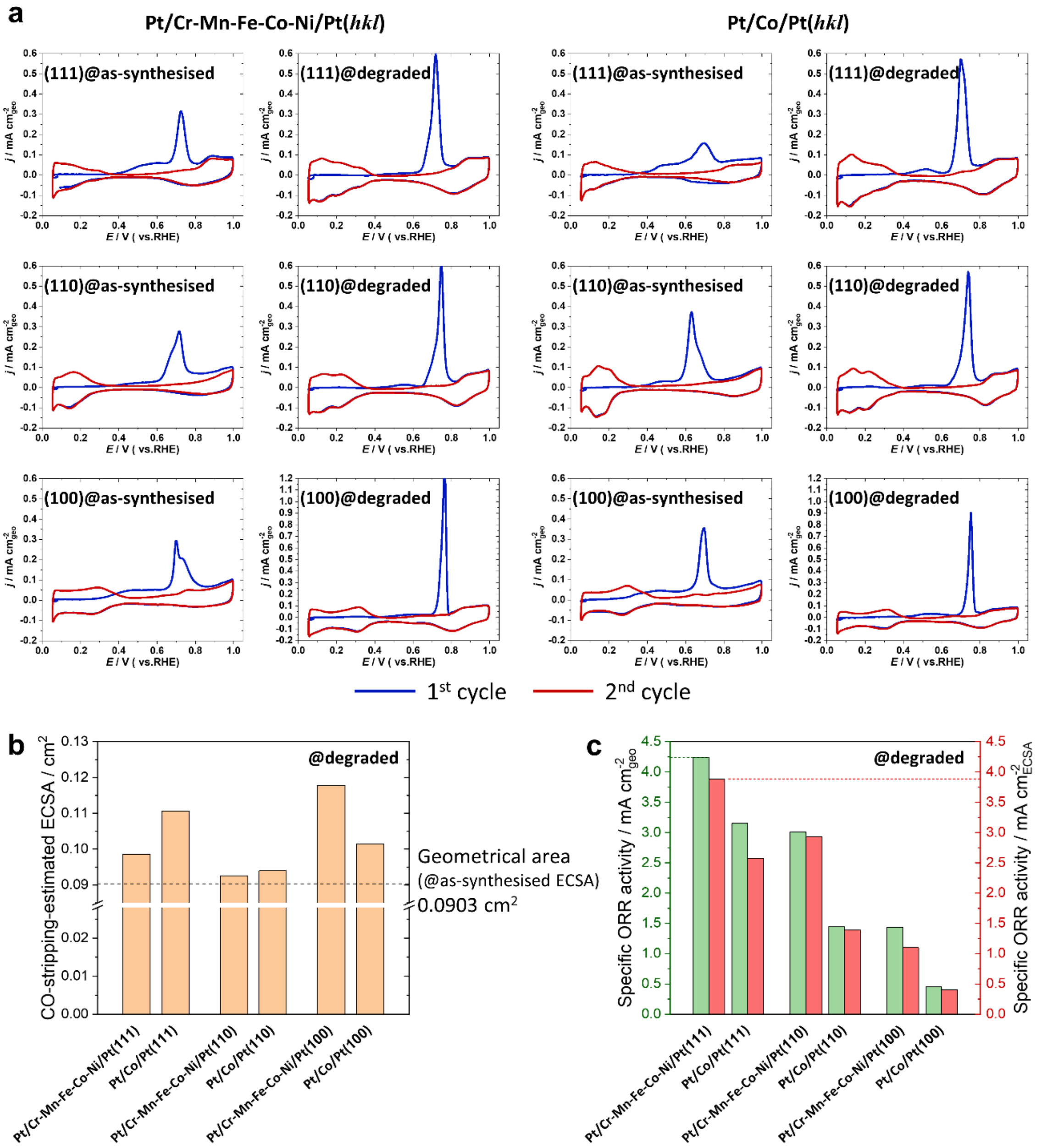

Fig. S8 a, CO stripping voltammetry with a scan rate of 0.05 V/s in 0.1 M $HClO_4$ at 25 °C for the as-synthesised and degraded (5,000 PC-loaded) Pt/Cr-Mn-Fe-Co-Ni/Pt(*hkl*) and Pt/Co/Pt(*hkl*) surfaces. The first CV cycle with adsorbed CO-oxidation (blue) and the subsequent (second) CV cycle (red) were displayed for the respective surfaces and states (as-synthesised (left panels) and degraded (right panels)). **b**, CO-stripping voltammetry estimated effective electrochemical surface area (ECSA) at the degraded state for each surface. The inset dashed line represents the geometrical ECSA for the O-ring confined working electrode surface

(see the main manuscript). **c**, specific ORR activities at the degraded state calculated from the geometrical surface area (left vertical axis, green; see also Figs. 3b, 3d and 3f) and effective ECSA (right; red) for each sample.

CO stripping voltammetry was conducted to estimate the effective electrochemical surface areas (ECSA) of the surface Pt layers for the as-synthesised (pristine) and degraded (5,000 PC-loaded) Pt/Cr-Mn-Fe-Co-Ni/Pt(*hkl*) and Pt/Co/Pt(*hkl*) surfaces. The recorded voltammetry curves are shown in Fig. S8a. The onset potentials of CO-oxidation stripping features are approximately 0.4 V vs. RHE on the positive-going sweep of the first CV cycle (blue lines), followed by the main peaks appearing at 0.6 ~ 0.8 V. The potentials of main peaks are located on the negative potential side compared to clean Pt surface[20–23]. The negative shifts probably stem from influences of the underlaid alloying metal elements[24]. These stripping current responses for the first potential sweeps were absent from the second CV cycle (red lines), indicating that the first positive-going sweeps completely oxidised the CO adsorbed on the Pt surfaces, irrespective of the surface orientation or of underlaid alloying elements. The CO-oxidation stripping charges that were estimated by subtracting the first curves from the corresponding second (background) curves in the potential region of 0.4–1.0 V, were nearly identical for all the as-synthesised samples, suggesting that the effective ECSA was almost equivalent to the geometrical area of each working electrode surface confined by the O-ring (0.0903 cm^2; main manuscript). Then, the effective ECSA for each degraded (5,000 PC-loaded) surface was calculated from the increase in the CO-oxidation stripping charge from the as-synthesised surface. The calculated values are summarised in b. Furthermore, specific ORR activities were estimated from the j_k value at 0.9V vs. RHE and the geometrical surface area (left vertical axis) or effective ECSA (right) for each surface. The results are shown in c. The increase in the estimated effective ECSA caused by PC-loading (5,000 PCs) is well correlated with the surface roughness of Pt/Cr-Mn-Fe-Co-Ni/Pt(*hkl*) (Figs. 4) and Pt/Co/Pt(*hkl*) (Figs. S5)

surfaces that appeared in the corresponding cross-sectional HAADF-STEM images. Furthermore, ORR deactivation was caused by applying PC-loading, i.e., the decreases in specific ORR activities calculated from the geometrical surface area (green) or from the effective ECSA (red) are qualitatively comparable to one another. The results emphasised the superior specific ORR activities of Pt/Cr-Mn-Fe-Co-Ni/Pt(*hkl*), relative to Pt/Co/Pt(*hkl*) benchmarks, even after 5,000 PC-loadings.

7. Atomic-level cross-sectional microstructure of the insufficiently heat-treated Pt/Cr-Mn-Fe-Co-Ni/Pt(111) surface prepared using a low post-annealing temperature

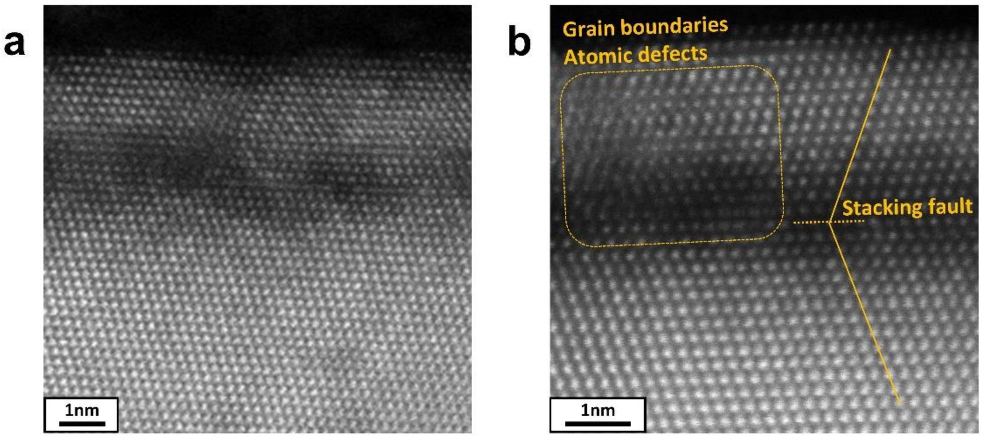

Fig. S9 a, b, Cross-sectional HAADF-STEM images at relatively low (a) and high magnifications (b) of Pt/Cr-Mn-Fe-Co-Ni/Pt(111) synthesised under the post-annealing temperature of 473 K for the surface Pt deposition.

The cross-sectional HAADF-STEM images of Pt/Cr-Mn-Fe-Co-Ni/Pt(111) synthesised at a post-annealing temperature of 473 K for surface Pt deposition are shown in figs. S9a and S9b. As shown clearly, the fabricated stacking structure include stacking faults and atomic defects, as compared to that at 623 K (Fig. 2a). This suggests that the thermodynamical conditions for the model catalyst sample synthesis, such as the post-annealing temperatures, strongly influence the resulting layer-by-layer and epitaxial stacking structures of the surface Pt and underlaid Cr-Mn-Fe-Co-Ni-rich lattices. That is, the post-annealing temperature for the synthesis of the Pt-HEA model catalyst is a critical experimental parameter for single-crystal epitaxial growth of the surface Pt(*hkl*) lattices on the corresponding Pt(*hkl*) substrates.

References

1. Cantor, B., Chang, I. T. H., Knight, P. & Vincent, A. J. B. Microstructural development in equiatomic multicomponent alloys. *Mater. Sci. Eng. A* **375–377**, 213–218 (2004).

2. Wadayama, T. *et al.,* Oxygen reduction reaction activities of Ni/Pt(111) model catalysts fabricated by molecular beam epitaxy. *Electrochem. Commun.* **12**, 1112–1115 (2010).

3. Tanuma, S. *et al.,* Experimental determination of electron inelastic mean free paths in 13 elemental solids in the 50 to 5000 eV energy range by elastic-peak electron spectroscopy. *Surf. Interface Anal* **37**, 833–845 (2005).

4. NIST Standard Reference Database 20, Version 4.1 http://dx.doi.org/10.18434/T4T88K

5. Lynch, D. W., & Olson, C. G. Photoemission Studies of High-Temperature Superconductors. (Cambridge University Press, 1999).

6. Briggs, D., & Seah, M. Practical Surface Analysis Second Edition Volume 1 – Auger and X-ray Photoelectron Spectroscopy. (John Wiley & Sons Ltd., 1996).

7. Wang, L. *et al.,* Study of the surface oxides and corrosion behaviour of an equiatomic CoCrFeMnNi high entropy alloy by XPS and ToF-SIMS. *Corros. Sci.* **167**, 108507 (2020).

8. Shen, X. *et al.,* Tuning electronic structure and lattice diffusion barrier of ternary Pt-

In-Ni for both improved activity and stability properties in oxygen reduction electrocatalysis. *ACS Catal.* **9**, 11431–11437 (2019).

9. Farkaš, B., Perry, C. B., Jones, G. & De Leeuw, N. H. Adsorbate-induced segregation of cobalt from ptco nanoparticles: modeling au doping and core auco alloying for the improvement of fuel cell cathode catalysts. *J. Phys. Chem. C* **124**, 18321–18334 (2020).

10. Nørskov, J. K. *et al.,* Origin of the overpotential for oxygen reduction at a fuel-cell cathode. *J. Phys. Chem. B* **108**, 17886–17892 (2004).

11. Greeley, J. *et al.,* Alloys of platinum and early transition metals as oxygen reduction electrocatalysts. *Nat. Chem.* **1**, 552–556 (2009).

12. Viswanathan, V., Hansen, H. A., Rossmeisl, J. & Nørskov, J. K. Universality in oxygen reduction electrocatalysis on metal surfaces. *ACS Catal.* **2**, 1654–1660 (2012).

13. Deshpande, S., Kitchin, J. R. & Viswanathan, V. Quantifying uncertainty in activity volcano relationships for oxygen reduction reaction. *ACS Catal.* **6**, 5251–5259 (2016).

14. Yeh, J. W. *et al.,* Nanostructured high-entropy alloys with multiple principal elements: Novel alloy design concepts and outcomes. *Adv. Eng. Mater.* **6**, 299–303 (2004).

15. Choi, J. *et al.,* Au-doped PtCo/C catalyst preventing Co leaching for proton exchange membrane fuel cells. *Appl. Catal. B Environ.* **247**, 142–149 (2019).

16. Yamada, Y. *et al.,* Oxygen reduction reaction activities for Pt-enriched Co/Pt(111), Co/Pt(100), and Co/Pt(110) model catalyst surfaces prepared by molecular beam

epitaxy. *Surf. Sci.* **607**, 54–60 (2013).

17. Kaneko, S. *et al.,* Ultrahigh vacuum synthesis of strain-controlled model Pt(111)-shell layers: surface strain and oxygen reduction reaction activity. *J. Phys. Chem. Lett.* **8**, 5360–5365 (2017).

18. Markovic, N., Hanson, M., McDougall, G. & Yeager, E. The effects of anions on hydrogen electrosorption on platinum single-crystal electrodes. *J. Electroanal. Chem.* **214**, 555–566 (1986).

19. Kusunoki, K. *et al.,* Oxygen reduction reaction of third element-modified Pt/Pd(111): Effect of atomically controlled ir locations on the activity and durability. *ACS Catal.* **11**, 1554–1562 (2021).

20. Ciapina, E. G., Santos, S. F. & Gonzalez, E. R. Electrochemical CO stripping on nanosized Pt surfaces in acid media: A review on the issue of peak multiplicity. *J. Electroanal. Chem.* **815**, 47–60 (2018).

21. Inkaew, P., Zhou, W. & Korzeniewski, C. CO monolayer oxidation at Pt(1 0 0) probed by potential step measurements in comparison to Pt(1 1 1) and Pt nanoparticle catalyst. *J. Electroanal. Chem.* **614**, 93–100 (2008).

22. Lebedeva, N. P., Koper, M. T. M., Herrero, E., Feliu, J. M. & Van Santen, R. A. Cooxidation on stepped Pt[n(111)×(111)] electrodes. *J. Electroanal. Chem.* **487**, 37–44 (2000).

23. Garcı´a, G. & Koper, M. T. M. Stripping voltammetry of carbon monoxide oxidation on stepped platinum single-crystal electrodes in alkaline solution. *Phys. Chem. Chem.*

Phys. **10**, 3802–3811 (2008).

24. Ochal, P. *et al.,* CO stripping as an electrochemical tool for characterization of Ru@Pt core-shell catalysts. *J. Electroanal. Chem.* **655**, 140–146 (2011)
