# Supplementary material for: Experimental study platform for electrocatalysis of atomic-level controlled high-entropy alloy surfaces
Source: Nat Commun. 2023 Jul 26;14:4492. doi: 10.1038/s41467-023-40246-5 (PMC10372069; doi:10.1038/s41467-023-40246-5)
Supplement: Supplementary file 3 — Figure dataset [file 41467_2023_40246_MOESM3_ESM.pptx]

## Slide 1
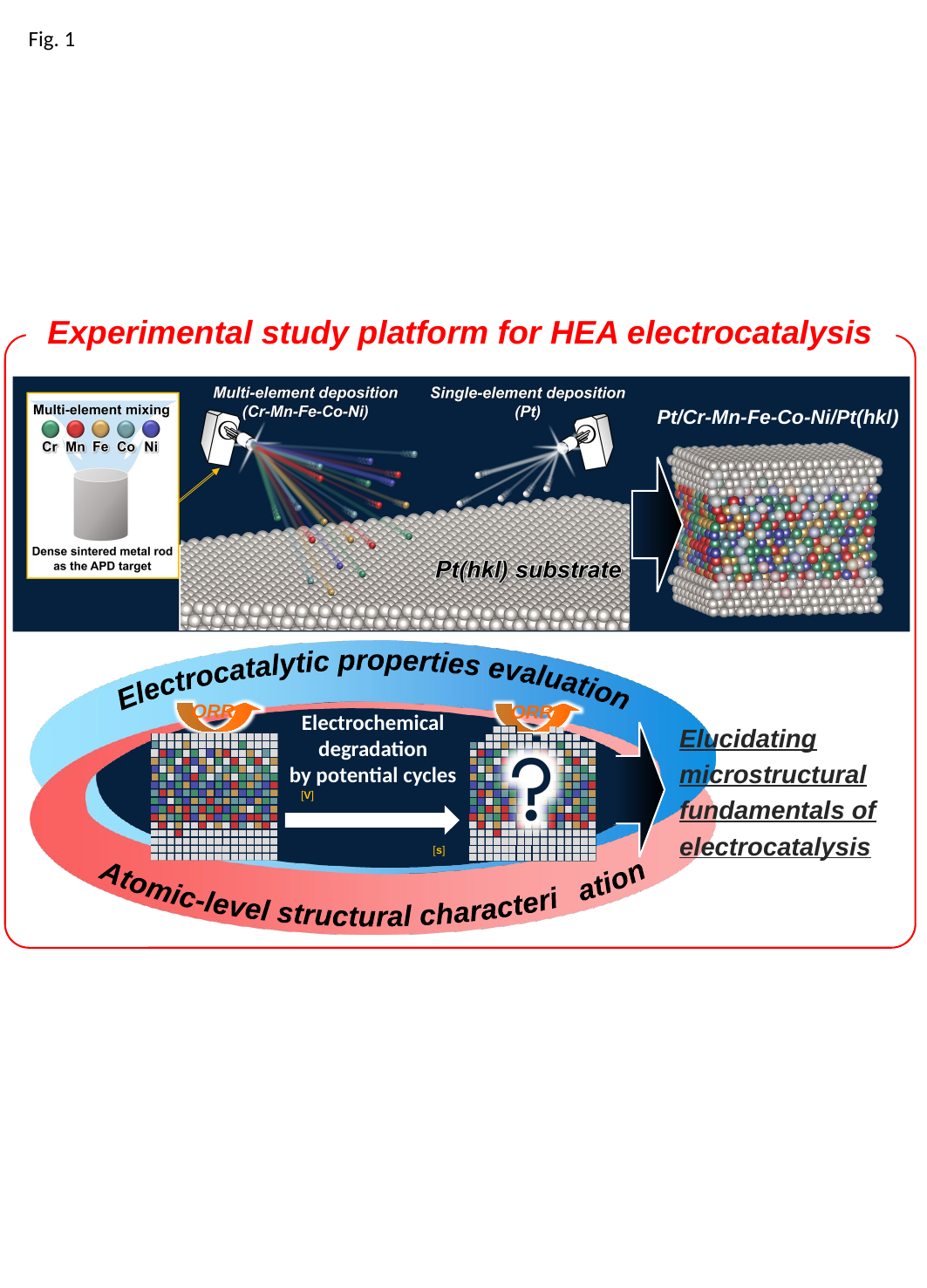

Fig. 1
Experimental study platform for HEA electrocatalysis
Pt/Cr-Mn-Fe-Co-Ni/Pt(hkl)
Electrocatalytic properties evaluation
ORR
ORR
Atomic-level structural characteriｓation
Electrochemical
degradation
by potential cycles
Elucidating
microstructural
fundamentals of
electrocatalysis
[V]
[s]

## Slide 2
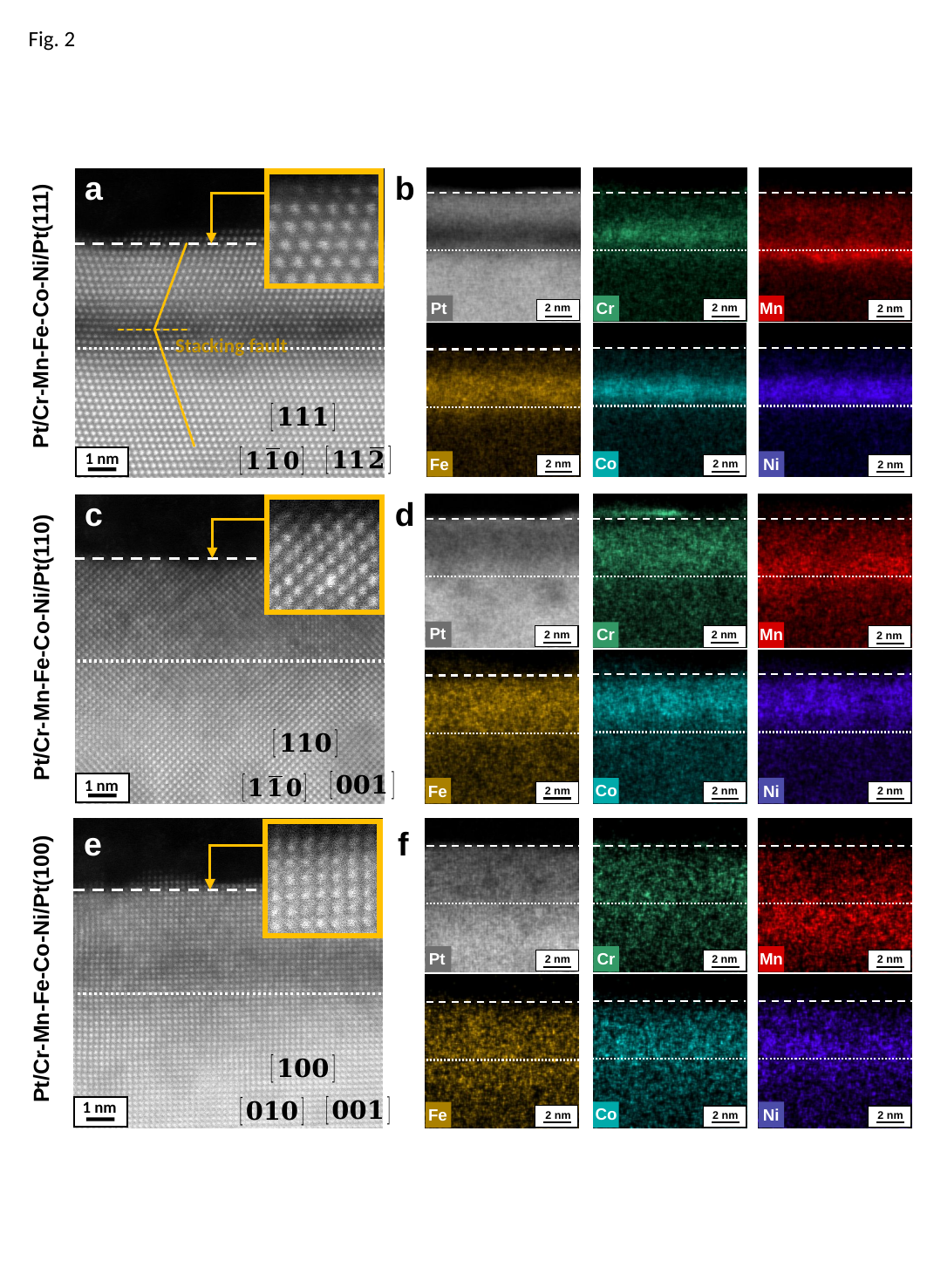

Fig. 2
b
a
Pt
Cr
Mn
2 nm
2 nm
2 nm
Pt/Cr-Mn-Fe-Co-Ni/Pt(111)
Stacking fault
1 nm
Co
Fe
Ni
2 nm
2 nm
2 nm
d
c
Pt
Cr
Mn
2 nm
2 nm
2 nm
Pt/Cr-Mn-Fe-Co-Ni/Pt(110)
1 nm
Co
Ni
Fe
2 nm
2 nm
2 nm
f
e
Cr
Pt
Mn
2 nm
2 nm
2 nm
Pt/Cr-Mn-Fe-Co-Ni/Pt(100)
1 nm
Co
Ni
Fe
2 nm
2 nm
2 nm

## Slide 3
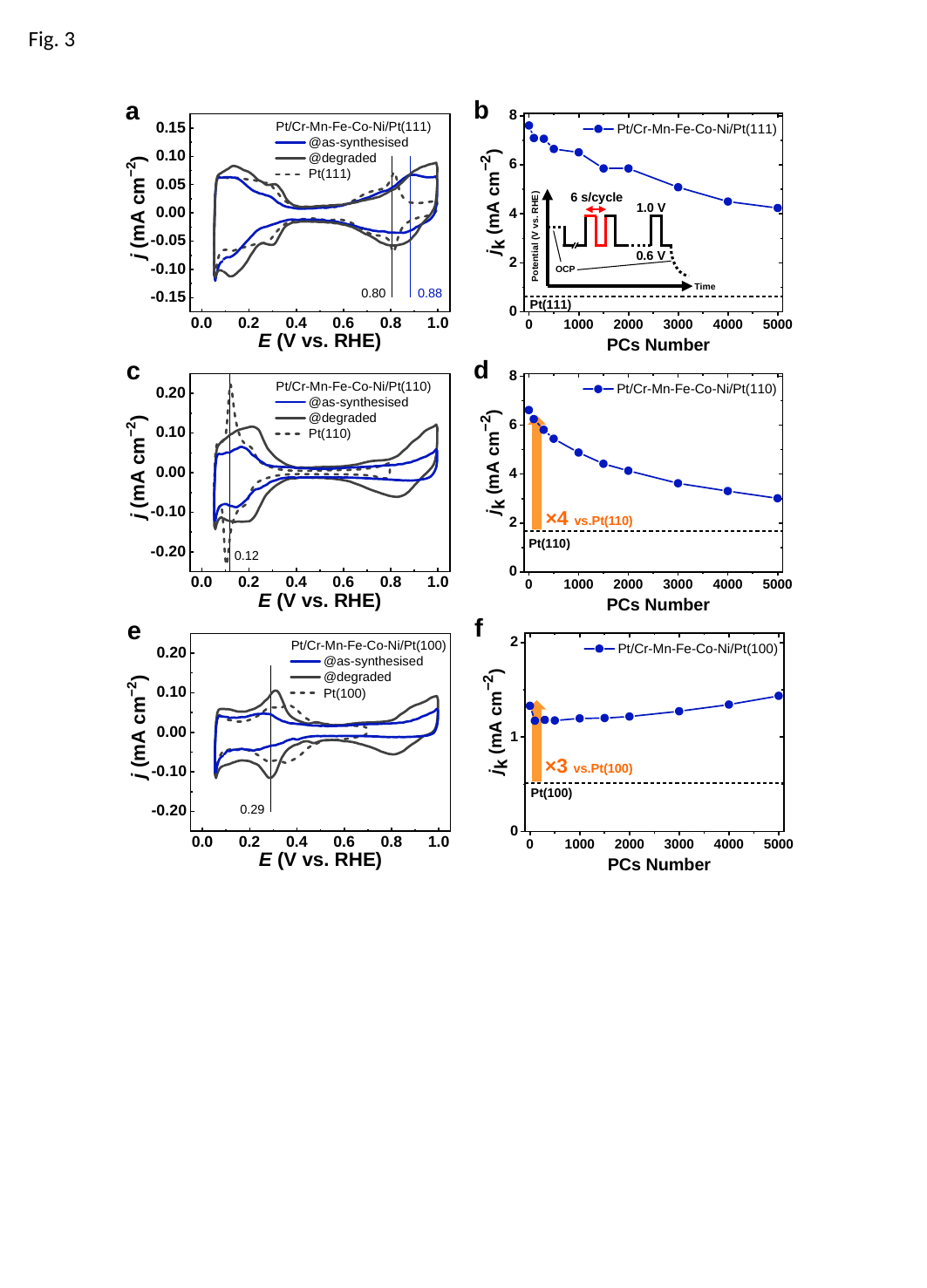

Fig. 3
0.88
0.80
b
a
6 s/cycle
1.0 V
Potential (V vs. RHE)
0.6 V
OCP
Time
d
c
×4 vs.Pt(110)
0.12
f
e
×3 vs.Pt(100)
0.29

## Slide 4
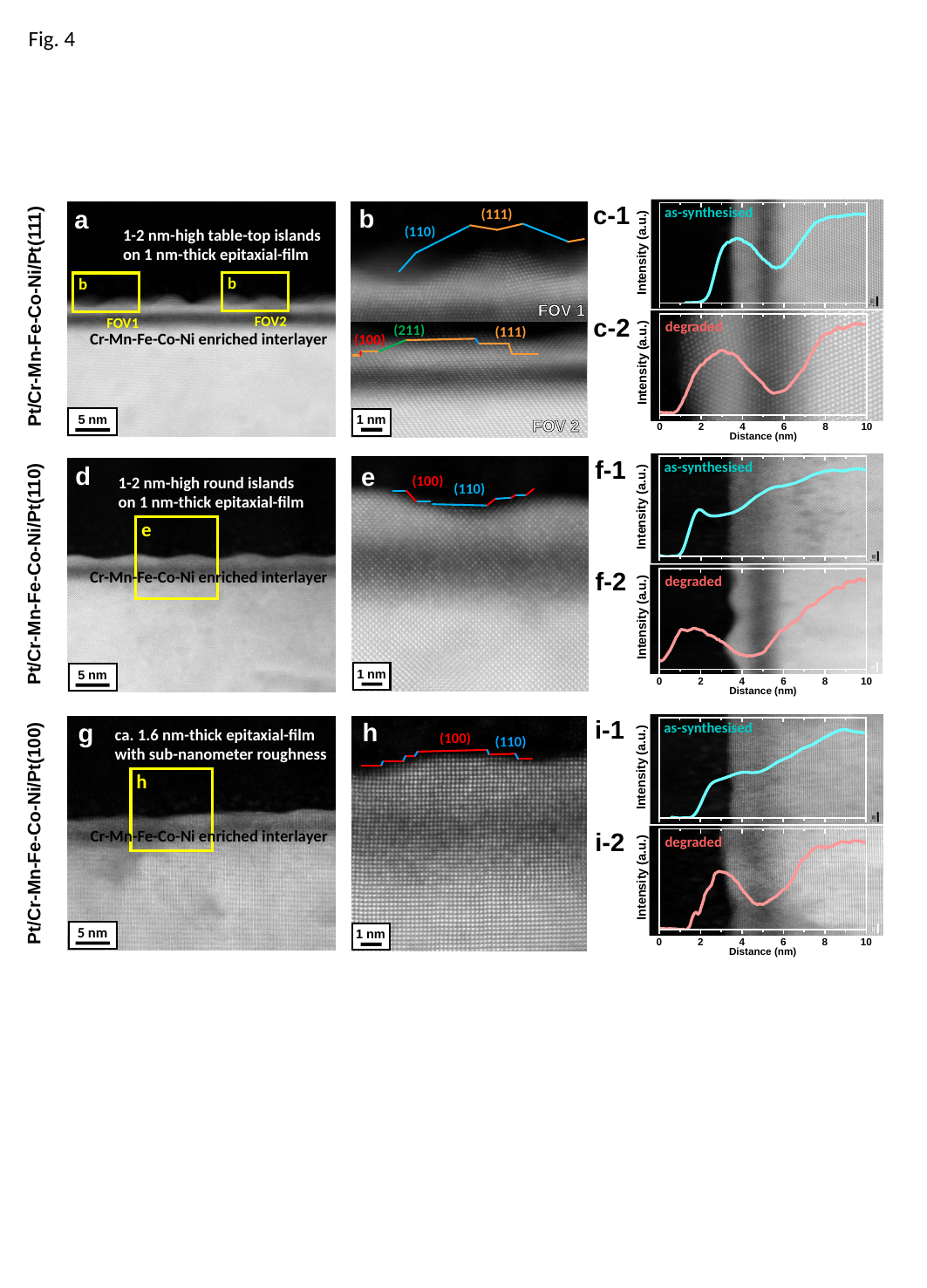

Fig. 4
c-1
d
b
(111)
(110)
FOV 1
(211)
(100)
1 nm
FOV 2
as-synthesised
a
1-2 nm-high table-top islands
on 1 nm-thick epitaxial-film
1~2 nm high islands
on 1 nm thin-film
Intensity (a.u.)
b
b
Pt/Cr-Mn-Fe-Co-Ni/Pt(111)
c-2
FOV2
FOV1
degraded
(111)
Cr-Mn-Fe-Co-Ni enriched interlayer
Intensity (a.u.)
5nm
5 nm
f-1
as-synthesised
d
1-2 nm-high round islands
on 1 nm-thick epitaxial-film
e
5 nm
e
(100)
(110)
1 nm
Intensity (a.u.)
Pt/Cr-Mn-Fe-Co-Ni/Pt(110)
f-2
Cr-Mn-Fe-Co-Ni enriched interlayer
degraded
Intensity (a.u.)
i-1
h
(100)
(110)
1 nm
g
as-synthesised
ca. 1.6 nm-thick epitaxial-film
with sub-nanometer roughness
Intensity (a.u.)
h
Pt/Cr-Mn-Fe-Co-Ni/Pt(100)
Cr-Mn-Fe-Co-Ni enriched interlayer
i-2
degraded
Intensity (a.u.)
5 nm
